# 

**Published:** 1965-04

**Authors:** 


					April 1965
\ "rational library of medicine
App- 6S"
[KCO- ii' AY 3 1535
/f 5 & K
~Qn^-?& V0L-1SSUE
Jzj 1NDEXER"
B INCORPORATING THE MEDICAL JOURNAL OF THE SOUTH WEST
~ u-l 80 (ii) No 296

				

